# Design and Implementation of a Fuzzy Classifier for FDI Applied to Industrial Machinery

**DOI:** 10.3390/s23156954

**Published:** 2023-08-04

**Authors:** Silvia Maria Zanoli, Crescenzo Pepe

**Affiliations:** Dipartimento di Ingegneria dell’Informazione, Università Politecnica delle Marche, 60131 Ancona, Italy; c.pepe@univpm.it

**Keywords:** fault detection, fault isolation, principal component analysis, statistical test, ANOVA test, industrial machinery, multishaft centrifugal compressor, cluster analysis, pattern recognition analysis, fuzzy C-means

## Abstract

In the present work, the design and the implementation of a Fault Detection and Isolation (FDI) system for an industrial machinery is proposed. The case study is represented by a multishaft centrifugal compressor used for the syngas manufacturing. The system has been conceived for the monitoring of the faults which may damage the multishaft centrifugal compressor: instrument single and multiple faults have been considered as well as process faults like fouling of the compressor stages and break of the thrust bearing. A new approach that combines Principal Component Analysis (PCA), Cluster Analysis and Pattern Recognition is developed. A novel procedure based on the statistical test ANOVA (ANalysis Of VAriance) is applied to determine the most suitable number of Principal Components (PCs). A key design issue of the proposed fault isolation scheme is the data Cluster Analysis performed to solve the practical issue of the complexity growth experienced when analyzing process faults, which typically involve many variables. In addition, an automatic online Pattern Recognition procedure for finding the most probable faults is proposed. Clustering procedure and Pattern Recognition are implemented within a Fuzzy Faults Classifier module. Experimental results on real plant data illustrate the validity of the approach. The main benefits produced by the FDI system concern the improvement of the maintenance operations, the enhancement of the reliability and availability of the compressor, the increase in the plant safety while achieving reduction in plant functioning costs.

## 1. Introduction

In the last decades, practitioners, engineers and researchers are focusing their attention on the study, development and implementation of technologies capable to guarantee defined levels of efficiency in industrial and non-industrial processes and machineries [1,2,3,4]. In this context, Industry 4.0 [5,6,7] and Maintenance 4.0 [8,9] are playing a crucial role, together with data selection, storage, and analysis [10,11,12]. Improvement of efficiency in industrial and non-industrial processes and machineries can be obtained in plants operation and maintenance operations. Examples of technologies for the improvement of plants operation are represented by Advanced Process Control (APC) systems [13,14,15,16] while maintenance can be optimized through FDI systems [17,18,19,20,21].

Great attention has been given to the detection and prevention of malfunctions and faults in machineries. These events can lead to downtime and shutdown and, in the worst cases, to their failure or possible breaking. Instrument single and multiple faults could cause errors in the data and/or in the operation of actuators. The detection and isolation of these faults represents a challenging problem that can be solved through Instrument FDI (IFDI) systems [22,23,24]. Instrument faults can cause serious problems to the machineries and to the involved processes, because a problem of a measurement or of an actuation device may result in abnormal behaviors in the connected processes. Another challenging problem to solve is the detection and isolation of the crucial faults that are associated to process faulty conditions that indirectly affect a high number of process variables (PVs), hereafter denoted as process faults. These fault conditions are not caused by faulty sensors or actuators as in IFDI, but are related to exogenous causes, such as the infiltration of water into a gear.

Examples of industrial machineries which attracted the attention of the FDI research in the last decades are machineries in industrial processes, manufacturing processes and theoretical processes ([25,26,27,28,29]), e.g., motors ([30,31,32,33,34,35,36,37,38,39]) and pumps ([40,41,42,43,44,45,46,47]). In [25], PCA is exploited together with the expectation formulas of T^2^ and squared prediction error statistics in order to achieve the detectability conditions of different faults in a simulated double-effective evaporator process. In [26], a fault diagnosis model based on signed digraph, support vector machine (SVM) and improved PCA method is applied to the Tennessee Eastman Process. Multiway PCA, recursive PCA, fault detection and Hotelling’s T^2^ statistic are used in [27] for the detection and diagnosis of process abnormalities in semiconductor manufacturing processes. A procedure for rotor fault discrimination in three-phase squirrel cage induction motors is presented and tested in [28], exploiting instantaneous active and reactive power signature analyses and their derived signals. In [29], a predictive maintenance model for the utilization of an injection molding machine is designed based on Machine Learning algorithms that are used for the distinction between borderline and correct operation.

With regard to motors, a radial-basis-function multilayer-perceptron cascade-connection neural-network-based fault detection scheme is developed in [30] for experimental fault detection in three-phase induction motors, exploiting PCA. In [31], fault detection experiments for different types of bearings in induction motors are reported, while Concordia transform (CT) and PCA are exploited in [32] for diagnosis tasks in three-phase electrical machines. In [33], an automatic algorithm based on an Hebbian-based unsupervised neural network is proposed for an online diagnostic of three-phase induction motor stator faults. The neural network is used to extract the PCs of the stator current data; the proposed procedure has been experimentally tested. An integrated approach for online experimental induction motor fault detection and diagnosis is presented in [34]. A three-step algorithm is used, based on data acquisition, eigenvector/eigenvalue computation, and the report of the extent of a fault. In [35], an induction motor simulator with normal, rotor failure, and bearing failure states is designed; the simulated data are exploited for failure diagnosis through different techniques, e.g., neural networks and machine learning models. In [36], fault diagnosis of electric motors is addressed. In particular, deep learning techniques are exploited, e.g., autoencoders and deep belief networks. In [37], PCA, squared prediction error (SPE) and Hotelling’s T^2^ statistics are used for the design of a module which detects vibration abnormal behaviors of a motor. In [38], a fast and accurate motor condition monitoring and early fault-detection system using 1-D convolutional neural networks is proposed and tested through tailored experiments. Electrical and mechanical faults in induction motors are presented in [39], together with their influence on the motor vibration in the frequency domain.

With regard to pumps, model-based fault detection for centrifugal pumps and AC drives is proposed in [40]; the role of parameters’ estimation in the diagnosis of pumps is investigated. A predictive maintenance procedure is developed in [41] based on signal analysis of pressure and differential pressure flow measurements. In [42], the design and initial testing of a quantitative model-based FDI scheme for a water pumping system is proposed. A centrifugal pump fault diagnosis method is proposed in [43] based on the contrast in vibration data obtained from a centrifugal pump under several operating conditions. Kurtogram images of time series vibration sequences and deep learning tool convolutional encoder are exploited. Multistage centrifugal pumps are considered in [44], where a fault diagnosis method is proposed using informative ratio PCA. In [45], an end-to-end pipeline for diagnosing faults in centrifugal pumps is proposed. Binary tree fast kurtogram is used; a convolutional autoencoder and convolutional neural network are trained to autonomously extract global and local features from the kurtograms. In [46], a three-stage lightweight framework for centrifugal pump fault diagnosis is proposed. Walsh transform and spectra are exploited and a K-nearest neighbor classifier is used. Centrifugal pumps are considered also in [47], where an automated health state diagnosis framework that combines a signal to time-frequency imaging technique and an Adaptive Deep Convolution Neural Network model is proposed.

A very challenging field where the mentioned FDI technologies could be applied is represented by the oil and gas treatment industry. Oil and gas treatments involve complex processes, e.g., energy production, refinery, gasification, and natural gas extraction/compression. Centrifugal compressors represent industrial machinery widely utilized in oil and gas treatment and their efficiency can significantly affect the efficiency of the overall processes [48,49]. Various control and supervision methodologies for centrifugal compressors were developed by researchers, engineers and practitioners. Examples of challenging topics are speed regulation [50], robust stabilization [51], and surge avoiding [52,53,54,55,56]. In addition to these topics, FDI systems on centrifugal compressors have also proven to be a very useful tool [57,58,59,60,61,62,63,64,65,66]. Time series analysis is combined with neural network in [57] to perform a correct fault diagnosis on centrifugal compressors. In [58,59], fault diagnosis on a centrifugal compressor is performed through the study of the vibration signals. A reliability study on a particular class of thermo-resistances used in crude oil extraction is proposed in [60], focusing on detect failures. In addition, a Failure Tree Analysis is performed in order to take into account maintenance as well as assembling operations. In [61], the faulty behavior of a centrifugal compressor is approached with a predictive maintenance assessment matrix taking into account detection techniques (capability and coverage), diagnosis (fault type, location, and severity), and prognosis (precision and predictive horizon). The proposed method is tested on a real centrifugal compressor. In [62], a machine’s health monitoring system is proposed for anomaly detection on a centrifugal compressor using an ensemble model, while in [63] an approach based on thermal parameters is studied; here, a qualitative simulation-based fault diagnosis method is designed to diagnose the faults of a centrifugal compressor. In [64], a dynamic process monitoring method based on canonical variable analysis and long short-term memory is proposed. The approach was evaluated using process data obtained from an operational industrial centrifugal compressor. A focus on the monitoring of radial vibrations of centrifugal compressors can be found in [65], where a smart model is developed using historical data based on essential parameters influencing rotor dynamics. A fault detection case study using this method is described, e.g., vibration variation due to abnormal opening of an anti-surge control valve. A strategy based on the method of hybrid Kernel-SVM method is adopted in [66]; here, a fault detection and localization strategy applied to a centrifugal gas compressor system is proposed which exploits experimental data.

To the best of the authors’ knowledge, considering the previously mentioned literature, several works have concerned the problem of fault diagnosis of single compressor components such as, for instance, the three-phase motor. On the other hand, a global and comprehensive FDI framework for a compressor unit is not widely cited in the literature. This paper proposes an FDI system for the detection and isolation of faults on a multishaft centrifugal compressor used for syngas manufacturing. The focus of this study is a multishaft centrifugal compressor used for nitrogen compression in the dilution of a particular synthetic gas, called syngas, which is forwarded to a gas turbine. The target of the developed system is to provide a tool for FDI approaches on compressors from a global point of view, i.e., taking into account all the crucial components. Multivariable data-driven, model-free procedures were applied for the system design, exploiting PCA. The authors selected a model-free approach motivated by the fact that in large-scale industrial plants the development of a reliable and tractable model is a challenging task; in fact, if a model can be achieved, it is often nonlinear and characterized by high dimensions. As mentioned in the previous, PCA is widely used in FDI systems. The approach is here customized so as to obtain a robust procedure that does not depend on subjective criteria to be applied to any real industrial process characterized by complex behavior. When approaching an FDI problem exploiting PCA, the number of PCs retained in the model is an essential parameter and ultimately determines its performance. Several methods for selecting the optimum number of PCs have been proposed in the literature [67,68,69]. The application of these methods typically suffers from restrictions: some of these methods are rather subjective, and other methods do not offer the possibility to work with both correlation and covariance matrix of the data. To overcome these difficulties, an approach based on the statistical test ANOVA (ANalysis Of VAriance) [70] is proposed by the authors. Another key point of the paper is represented by the number of variables involved in the process faults analysis. Usually, if the considered process is complex, a growth of complexity in the analysis can be observed. In order to mitigate this problem, the authors adopt an automatic procedure based on Cluster Analysis for the isolation of the main known faults. A Fuzzy Faults Classifier is proposed to perform the detection of the system fault prototypes. In addition, in order to provide more comprehensive information, the output of the FDI system is provided in terms of fault probability, and the faults characterized by a higher probability are notified by an automatic online Pattern Recognition procedure.

The paper is organized as follows: Section 2 reports the material and methods, providing the plant description (motivating also the need of an FDI system for the considered case study), a background on the PCA, some details on the exploited FDI method, the computational architecture of the developed framework, and a comparison between the proposed FDI framework and other procedures. Section 3 reports the results and discussion, while the conclusions are summarized in Section 4, together with some ideas for future work.

## 2. Materials and Methods

### 2.1. Plant Description

**Figure 1:**
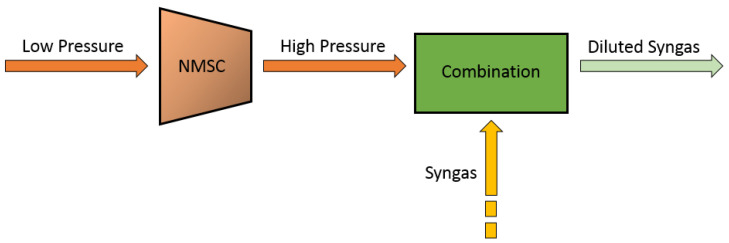
Plant overview.

In the present work, a Nitrogen MultiShaft Compressor (NMSC) system located in a unit for the syngas manufacturing is considered. The NMSC is utilized to compress the nitrogen in the process of the syngas dilution. This complex machinery is composed of two separate sections where compression takes place: in the first part, two compression stages take place, while the second part is constituted by three compression stages. Direction vanes, called Inlet Guide Vanes (IGV), adjust the nitrogen mass flow through the suction in the first three compression stages. A three-phase asynchronous motor drives the compressor. The transmission system transmits the torque from the engine to the compressor. The compressor is multishaft and the coupling between it and the transmission shaft of the motor is characterized by three joints. The engine speed is about 1500 rev/min, while the compression shaft speeds are about 15,000 rev/min, thus providing transmission ratios equal to 10. The first and the second compression stages are coupled through the first joint, and the second joint couples the third stage while the third joint couples the fourth and the fifth stage. Each compression stage is followed by a heat exchanger at its end. The heat exchanger is constituted by a shell side and a tube side: in the shell side, a hot fluid, i.e., nitrogen (N_2_), is circulated. N_2_ is supplied from the process, while in the tube side, cold liquid, i.e., sea water, is forced through. The heat exchanger aims to decrease the N_2_ temperature at the exit of the compression stage; in this way, a leveling between the N_2_ temperature at the begin and at the exit of the compression stage is obtained. This allows near-isothermal operation of the compressor, which increases the efficiency by 17% to 30% compared to adiabatic compression [49]. Figure 1 reports an overview of the considered plant.

#### The Need of an FDI System on NMSC

The compression process can be subjected to single and multiple faults which could be the cause of errors in the sensors data and/or in failure on the actuators. Furthermore, the detection of the process faults caused by disturbances entering the process from the environment through one or more exogenous (independent) variables is another key and crucial objective to be targeted. In this way, serious breaks on the plant could be prevented and avoided so as to contribute to efficiency and safety improvement. A feasibility study on the considered process, based on a thorough data selection, acquisition, storage and analysis procedure ([10]), revealed that different compressor process faults were present. The criticality of the process faults is represented by the fact that many variables are interested and the identification of these faults is a very difficult and challenging problem to solve.

Among the most severe causes of faults in NMSC, there is the fouling of the compression stages, together with breaks of thrust bearing. Sea water leakage in the heat exchanger and the consequent sea salt deposit is a typical cause of fouling. When the NMSC is in Normal Operative Conditions (NOC), the water pressure is lower than the N_2_ pressure: in these conditions, the sea water cannot enter the shell side of the exchange. During shut-down periods, a reduction in the gas pressure takes place and the water pressure overcomes the N_2_ pressure: in this condition, water can leak from the side of the shell. This fact causes a deposit of sea salt in the NMSC frame. This condition causes changes in the heat transfer coefficient and, if it is not promptly detected, an efficiency decrease is registered. This efficiency decrease is coupled to horizontal and vertical shaft vibrations increase and thrust bearing temperature increase. Furthermore, in order to equalize the N_2_ flow in the NMSC, IGV opening is increased; in this state, the compressor and the downstream equipment do not work in optimal conditions: mechanical parts wear out more easily.

Other conditions in NMSC that preferably require early detection include malfunctions in lube oil systems and not optimal calibration of rotor shaft; in fact, these malfunctions can damage thrust bearing (see [31] for other faults associated to different types of bearing) and can cause its complete break. This break can cause the shut-down of the plant.

The most common NMSC faults that have been considered for the development of the FDI system are reported in Table 1. The considered faults are associated to both sensors/actuators and process. The instrument faults that may possibly occur in the NMSC concern errors in the sensor readings and/or failures of the actuators. In Table 1, historical faults of the compressor and their time dependency are summarized. In order to perform FDI on the NMSC, the process variables reported in Table 2 are considered. As this process is very critical, measurement accuracy is a key requirement and is ensured in the proposed case study. In addition, tailored logics have been implemented in order to detect, preprocess and (eventually) discard bad input data. Some of the performed checks take into account compliance with validity limits, and rate of change and freezing checks. These logics that ensure the robustness of input data are always implemented in critical plants such as those for syngas production. The exploited data acquisition and storage architecture are reported in Section 2.5.

### 2.2. Background on the PCA

Within the designed FDI system, Principal Component Analysis was adopted [18,28]. PCA is performed by a mathematical procedure which transforms a set of possibly correlated variables into a set of Principal Components, i.e., a smaller number of uncorrelated variables. A subspace decomposition is performed within PCA: the process measurement space is divided into two orthogonal subspaces. These two subspaces are the PC subspace and Residual subspace. As mentioned in Section 1, PCA is widely used in FDI problems due to its efficiency and simplicity to handle huge amount of data. This fact elected PCA as a powerful tool for statistical process monitoring.

Considering a matrix $X\in R^{N\times n}$, where *N* is the number of data samples and *n* is the number of variables, a transformation of the possibly correlated variables into a smaller number *l* < *n* of uncorrelated variables is performed. The transformation executed by PCA is linear and divides the process measurement space into the two previously mentioned orthogonal subspaces. The main steps of PCA are summarized in the following [18,28].

The first step consists in the computation of the following matrix *A* from the original matrix *X*:

(1) $$A_{\left[n\times n\right]}=X^{T}{}_{\left[n\times N\right]}X_{\left[N\times n\right]}.$$

Matrix *A* is proportional to the covariance matrix associated to the original data if *X* is scaled to the zero mean; on the other hand, if *X* is scaled to the zero mean and to unit variance, the matrix *A* is proportional to the correlation matrix associated to the original data.

The second step performs the computation of the Loading Matrix *P* starting from the *l* eigenvectors derived through the selection of the *l* most significant eigenvalues of the matrix *A* (see in the following):

(2) $$P_{\left[n\times l\right]}=\left[\begin{array}{cccc} p_{1} & p_{2} & \ldots & p_{l} \end{array}\right].$$

The third step is the computation of the Score Matrix *T*:

(3) $$T_{\left[N\times l\right]}=X_{\left[N\times n\right]}P_{\left[n\times l\right]}.$$

The matrix *T* includes information of the original data but it is represented by a smaller number of variables *l* < *n*. These variables are the PCs and they provide about the same information with respect to the variance of the original data.

The fourth step is represented by the computation of the Back-Transformation. This transformation allows return to the original data space.

(4) $$X^{*}{}_{\left[N\times n\right]}=T_{\left[N\times l\right]}P^{T}{}_{\left[l\times n\right]}=X_{\left[N\times n\right]}P_{\left[n\times l\right]}P^{T}{}_{\left[l\times n\right]}.$$

Through the Back-Transformation process, the original variables can be obtained without non-significant noise effects, i.e., only with the significant information on variance.

Subtracting the reconstructed data to the original data allows the computation of the residuals. The residual analysis allows the detection of possible faults through a comparison of the current residuals with the residuals in NOC (no fault).

A reliable FDI system must ensure not only fault detection but also fault isolation. For this purpose, different methods based on the generation of the residuals were developed in the literature. In the present work, the method reported in [21] was applied, i.e., the structural residual approach. As mentioned in Section 1 and as it is explained in the following, this method was enriched with an innovative statistical method in the present work. The overall procedure allows the selection of the “best” number *l* of eigenvalues, i.e., the number of PCs that can explain the maximum variability of the data set. Exploiting PCA, the original matrix *X* can be decomposed as

(5) $$X=X^{*}+\tilde{X},$$

(6) $$X^{*}=XC_{l},$$

(7) $$\tilde{X}=X\left(I-C_{l}\right),$$

(8) $$C_{l}={\left(P^{T}P\right)}^{-1}=PP^{T},$$

where $X^{*}$ and $\tilde{X}$ are the projections of *X* into the PCs subspace and Residual subspace, respectively.

Exploiting Equation (1), subset *R* can be estimated; this subset contains the reconstructed variables indices. The reconstruction procedure aims to estimate a matrix ${\hat{X}}_{R}$ which includes the faults’ effect. Subsequently, a new projection matrix allows projection of the reconstructed variables on the Residual subspace (see [21] for further details). The isolation of potential faults takes place due to a property of the projection matrix: the product of the projection matrix with the reconstruction directions matrix is null. This property ensures that the residuals (computed through the projection matrix), at defined time instants, are significant only for a set of the overall PVs: comparing the residuals’ behavior with their behavior in NOC, it is possible to identify fault directions. 

For large data sets, the checking of all the single computed residuals could be not tractable. In order to solve this problem, a grouping policy of the residuals into a unique index can be performed. This index is named as Square Prediction Error (SPE) and it is computed through the Euclidean norm of the computed residuals (grouped in the ${\tilde{x}}_{R}$ vector):

(9) $$SPE_{R}=\|{\tilde{x}}_{R}\|{}^{2}.$$

Exploiting the SPE reported in Equation (9), fault isolation can be achieved by comparing its values to some thresholds in NOC (for the interested reader, approaches to SPE analysis are reported in [18]).

### 2.3. PCs Selection

One of the main issues to solve using a PCA approach is the selection of the PCs to be included in the model. This phase represents a crucial aspect and seriously affects the PCA algorithm performance. If an excessively low number of PCs is considered, the achieved model does not take into account all the available information from original data: a poor representation of the process is obtained in this case. If an excessively large number of PCs is retained instead, over-parametrization could take place and undesired noise may be maintained.

The investigation on the best number of PCs to be considered can be tackled through different methods. Typical methodologies are the scree test on Residual Percent Variance (RPV), Average Eigenvalue (AE), Minimum Description Length (MDL), Cumulative Percent Variance (CPV), Variance of the Reconstruction Error (VRE), cross-validation based on the PREdicted Sum of Squares (PRESS), Imbedded Error Function (IEF), Autocorrelation (AC), Parallel Analysis (PA), and Akaike Information Criterion (AIC). Some methods require the definition of an arbitrary “threshold”, introducing a degree of freedom in the algorithm design. For example, the CPV method requires to establish the desired CPV, e.g., 90%, 95%, or 99% [71]. This degree of freedom introduces a subjective (and not objective) aspect in the algorithm. AE [72] and PA [73] methods may depend on the number of the samples of the exploited dataset. Other methods depend on heuristics or rules. In the scree test on the RPV method [74], for example, the optimal number of PCs is computed taking into account the “knee” in the relative index trend. The main problem in this method is represented by the difficulty, in some cases, of correctly identifying the “knee”. The AC method includes an autocorrelation function of the PCs [75]: a threshold equal to 0.5 is imposed and autocorrelation values lower than the threshold are a symptom of noise presence in the component; thus, the considered component should be discarded and not included in the PCA model. In addition, other methods rely on the covariance or the correlation matrix, e.g., AIC [76], MDL [77], and IEF [78]; other approaches, e.g., VRE [79] or PRESS [80], are characterized by an almost monotonic decreasing behavior in some cases. Under these conditions, it is difficult to find a minimum point and consequently the choice of the number of PCs may not be adequate.

Due to the previous discussion, in this work, the ANOVA statistical test is used for the selection of the PCs in order to achieve the PCs subspace. This method is very reliable and can be applied to both correlation and covariance matrices. In addition, the uniqueness and objectiveness of the results represent additional pros. As reported in [70], ANOVA consists of a collection of statistical models; due to this feature, it is possible to compare two or more samples of a population through the comparison of the variance between the samples and within every sample. In other words, the ANOVA test allows the determination of whether an additional eigenvalue (i.e., the inclusion of an additional PC) could add value to the already available information. Because of the statistic nature of the ANOVA test, two assumptions, i.e., H_0_ and H_1_, are made on the model parameters and, in particular, on the correlation matrix eigenvalues. Hypothesis H_0_ assumes that the added eigenvalue can be neglected while assumption H_1_ establishes that the added eigenvalue is needed in the model. 

In order to apply the ANOVA test, three preliminary assumptions must hold:

1. A normal distribution characterized by a zero mean and variance *σ*^2^ must represent the reconstruction errors; if this assumption is satisfied, the denominator and the numerator of the statistical index *F* can be represented through a χ^2^ distribution as defined by Fisher’s test [70].
2. The reconstruction errors must be independent.
3. The variance of the reconstruction errors should be the same (*homoscedasticity* property).

In the present work, the first assumption is checked exploiting the KS Test (Kolmogorov–Smirnov Test) and inspecting the error histograms. The second assumption is checked using the KS Test on the error joint probability density function. Subsequently, the cross-correlation between the reconstruction errors is computed. The motivation of this procedure relies on the fact that, for Gaussian distributions, the uncorrelation implies independence [81]. On the other hand, if the reconstruction errors cannot be represented through a normal distribution, the results of the cross-correlation test must not be considered. In order to check the third assumption, Bartlett’s Test is used.

#### 2.3.1. Index of Reconstruction Error

In order to perform the ANOVA test, the previously reported assumptions must hold. These assumptions are associated to the statistical properties of the reconstruction errors. On the other hand, PCA requires to compute a large number of reconstruction errors; from a computational point of view, these calculations could be demanding. In order to limit the computational burden, an index vector is defined as the sum of the square errors of every model. To better clarify, considering the original matrix *X* (see Equation (1)), two reconstruction error indexes associated to two different models can be defined as follows:

(10) $$E_{1}=X-{\overset{̑}{X}}_{1}=\left[\begin{array}{cccc} e_{11}\left(1\right) & e_{12}\left(1\right) & \cdots & e_{1n}\left(1\right)\\ \vdots & \vdots & \ddots & \vdots \\ e_{11}\left(N\right) & e_{12}\left(N\right) & \cdots & e_{1n}\left(N\right) \end{array}\right]=\left[\begin{array}{cccc} e_{11} & e_{12} & \cdots & e_{1n} \end{array}\right],$$

(11) $$E_{2}=X-{\overset{̑}{X}}_{2}=\left[\begin{array}{cccc} e_{21}\left(1\right) & e_{22}\left(1\right) & \cdots & e_{2n}\left(1\right)\\ \vdots & \vdots & \ddots & \vdots \\ e_{21}\left(N\right) & e_{22}\left(N\right) & \cdots & e_{2n}\left(N\right) \end{array}\right]=\left[\begin{array}{cccc} e_{21} & e_{22} & \cdots & e_{2n} \end{array}\right],$$

where ${\overset{̑}{X}}_{1}$ and ${\overset{̑}{X}}_{2}$ are the estimation matrices which contain the reconstruction variables. In order to limit the computational burden, the check of the previously mentioned assumptions on every single column pair of $E_{1}$ and $E_{2}$ is replaced by the computation of the following index vectors:

(12) $$E_{1_{index}}=\left[\begin{array}{cccc} \|e_{11}\|{}^{2} & \|e_{12}\|{}^{2} & \cdots & \|e_{1n}\|{}^{2} \end{array}\right],$$

(13) $$E_{2_{index}}=\left[\begin{array}{cccc} \|e_{21}\|{}^{2} & \|e_{22}\|{}^{2} & \cdots & \|e_{2n}\|{}^{2} \end{array}\right],$$

where ‖.‖ denotes the Euclidean norm of the vector. Exploiting Equations (12) and (13), the required number of checks is limited. The definition of the Gaussian distribution does not change for the new index error vectors as can be seen in the first hypothesis in Section 2.3, while the other two hypothesis of Section 2.3 must be reformulated based on the following statements:

1. If errors associated to variables must be represented by a Gaussian distribution with a zero mean and variance *σ*^2^, the sum of the squared errors must follow a χ^2^ distribution with *n* degrees of freedom (where *n* is the number of the error vectors composing the sum).
2. If errors associated to each model are characterized by the same variance, the sum of the squared errors exhibit the same variance.

### 2.4. Fuzzy Faults Classifier (FFC)

**Figure 2:**
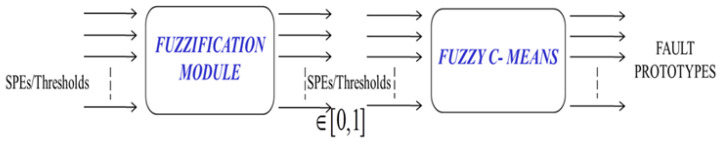
FFC: fault prototype computation.

**Figure 3:**
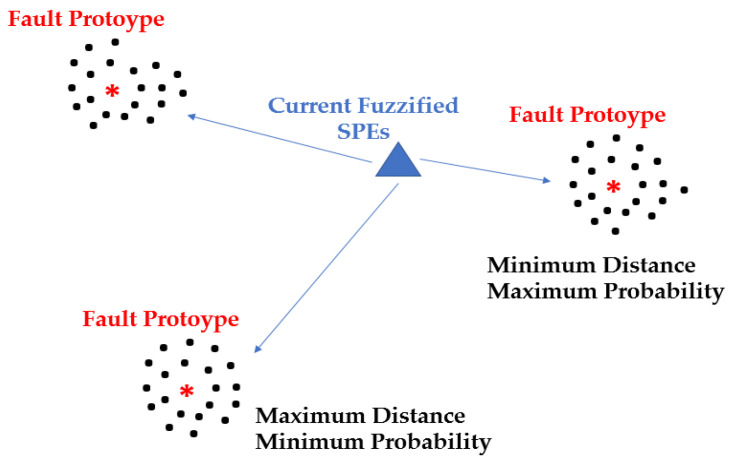
FFC: fault probabilities are inversely proportional to fault prototype distances.

In the previous sections, PCA and SPEs procedures were briefly described, highlighting the main aspects and key points. In practice, the major problem is that, often, a high number of SPEs needs to be investigated for process faults isolation due to the fact that process faults are associated to a high number of PVs. In these cases, different SPEs can exceed their threshold and uncertainty in the fault identification process can arise. In order to solve this problem, an automatic procedure for SPE inspection is developed which can provide an indication of the type of fault identified, at least within a known fault case history. The developed approach is based on Cluster Analysis and Pattern Recognition and involves Fuzzy inference [82,83]. The procedure, called Fuzzy Faults Classifier (FFC), is composed of two phases: the first, performed offline, computes the fault prototypes, while the second, performed online, computes the probability of occurrence of each fault at each sampling instant. Offline, after the computation of SPEs resulting from the application of the previously proposed PCA-based procedure, two computation steps are performed (see Figure 2). Fuzzification of the computed SPEs is the first step [84,85]; in this way, the comparison of the SPE values with their associated thresholds is performed in a fuzzy, not crispy, way. In the second step, fault prototypes are created through the application of Cluster Analysis techniques [86,87]. Online, after the fuzzification of the newly computed SPEs, a classification step is performed. To accomplish the classification task, the distances of the configuration associated with the current fuzzified SPEs to the defined fault prototypes are computed and a probability value is assigned to each prototype fault; a higher probability corresponds to a lower distance (see Figure 3). The current probability of each fault prototype is given as the output of the FDI system. The paradigm of the proposed FFC is to provide the most probable faults within records of known faults. In Figure 3, clusters’ centroids are represented by the * symbol.

#### 2.4.1. Fuzzification

**Figure 4:**
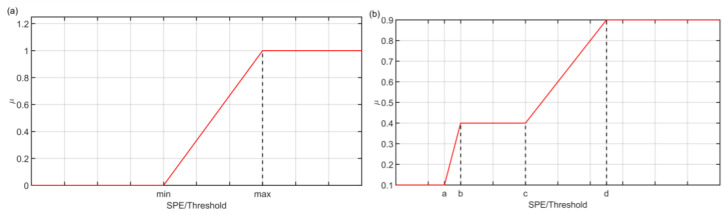
FFC: membership functions of the fuzzification module (**a**,**b**).

Different operating conditions characterize the exploited datasets: NOC and fault conditions were included in order to provide all the needed information to the FDI system. As explained, the first step of the proposed FFC is represented, both offline and online, by a fuzzification procedure where SPE values are compared to the defined thresholds in NOC; Figure 4 reports some examples of the adopted Membership Functions. The fuzzification module processes the SPEs computed through PCA and the thresholds defined in NOC. The defined thresholds could be fixed or adaptive. The decision to consider both fixed and adaptive thresholds was motivated by the fact that, in some cases, such as process faults that cause the variation of numerous variables, it has been experimentally observed that adaptive thresholds provide less satisfactory results and therefore the use of fixed thresholds, although more conservative, is preferable. In fact, in the case of process faults, considering adaptive thresholds, the simultaneous variation of several quantities results in the construction of thresholds that poorly follow the considered signals. In this case, fixed thresholds are adopted since they resulted the more effective.

#### 2.4.2. Cluster Analysis Procedure

The second step of the proposed offline FFC algorithm is represented by the creation of fault prototypes through Cluster Analysis. In particular, Fuzzy C-Means algorithm was exploited [88]. The minimization of the objective function of the Fuzzy C-Means algorithm provides the computation of the centroids. Specifically, for each process condition, including NOC and faulty situations, the residuals and associated SPEs are calculated. Of course, in order for the clustering algorithm to recognize the different classes, it is necessary to use a dataset containing at least two different situations (e.g., a no-fault condition and one containing the fault under consideration). If this is achieved, the residuals and consequently the SPEs will have a different pattern depending on the situation under consideration, and therefore it will be possible to identify the different centroids (one for each condition), calculate the values of the matrix containing the Membership Function degrees for each sample, and estimate the minimum of the objective function employed in the algorithm. Table 3 reports the defined fault prototypes, including instrument single faults and the two previously described process faults, i.e., the fouling of the BLNC first stage and the breaking of the thrust bearing of the BLNC first stage; in addition, multiple faults are included considering all variable combinations, taken two at a time, of the just-mentioned instrument single faults. In order to validate the consistency of the obtained clusters, the Jeffreys–Matusita (JM) distance between distributions is used [89]. This choice is motivated by the fact that other validation approaches (e.g., [90]) strictly depend on the process knowledge and no validation of the classification process outcome is provided. Therefore, in order to avoid misclassification issues, JM distance is used as a metric to validate the fault prototype generation procedure. In fact, once the clusters of prototypes are computed, their fairness can be argued by comparing the distributions related to each cluster. Consistency of the computed clustering is then inferred if they result sufficiently separated. In Section 3, examples of the procedure are discussed.

#### 2.4.3. False Alarms and Chattering Avoidance

False alarms represent a crucial issue in FDI systems [17] and they should be avoided. In order to smooth false alarms, a tailored filter was included in the proposed approach. For this purpose, a suitable filter was designed. Consider a dataset of *n* variables and assume that at the current instant the calculated SPEs (on one direction) exceed the defined threshold for *m* variables, where *m* ≤ *n* − 2. In this condition, a false alarm condition arises and the fault must not be reported.

An additional issue to be prevented in FDI systems is the chattering condition on the most probable reported fault. In this case, a filter was designed which, taking into account the past FFC outputs, suitably modifies the current FFC output.

### 2.5. FDI Framework Computational Architecture

**Figure 5:**
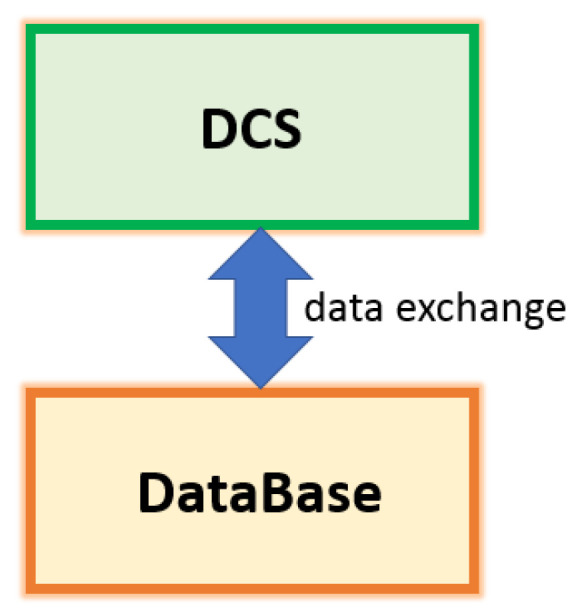
FDI Framework Computational Architecture: data exchange between DCS and database.

The architecture used for data acquisition and storage is reported in Figure 5. All data associated to the selected PVs are acquired on the real plant through a Distributed Control System (DCS) at the defined sampling time (see green rectangle in Figure 5). The acquired data are stored in a tailored database (see orange rectangle in Figure 5).

A MATLAB environment was used for the design of the FDI framework and for the computation of the results (see Section 3) [91].

**Figure 6:**
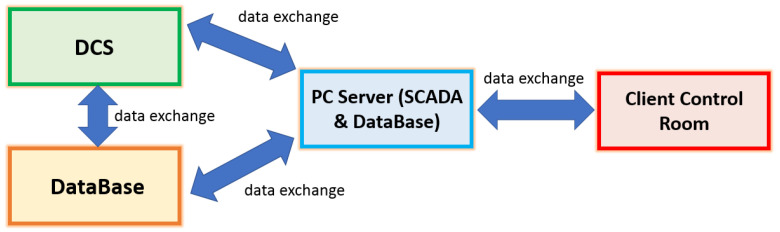
FDI Framework Computational Architecture: data exchange scheme for real-time implementation.

Figure 6 shows the designed architecture for real-time implementation. The software associated to the developed FDI system can run on a Supervisory Control And Data Acquisition (SCADA) system (see blue rectangle in Figure 6) which can be installed into an industrial PC server located on the plant. Because of the options provided by the adopted SCADA system, plant operators could monitor the proposed software through a client PC installed in the control room (see red rectangle in Figure 6). In the proposed architecture, plant information (e.g., sensors measurements and plant signals) are provided to the FDI system by plant DCS and a database. On the other hand, the FDI system sends the computed outputs to the plant DCS and the database and to the client PC (see Figure 6).

### 2.6. Comparison between the Proposed FDI Framework and Other Procedures

The crucial materials and methods of the proposed FDI framework were reported in the previous sections. The proposed FDI framework can be classified as a tool which considers the most significant components of a compressor. The applied procedure is multivariable, data-driven, and model-free: it is capable to take into account and to process many PVs and, in addition, it is strictly related to data and it does not depend on a process model. For this reason, reliable data represent the main requirement for a profitable application of the proposed method. No process model is required: this feature could represent an advantage with respect to model-based approaches, mainly in case studies where the formulation of a reliable process model is difficult to obtain. Based on the previous considerations, the proposed approach can be classified as a holistic approach. Some cons of the proposed method could be the need of reliable data, but also model-based approaches could require reliable data for the validation of the model to be used. With regard to the need of reliable data, the Industry 4.0 paradigm certainly represents a driver; thus, this issue can be solved in a straightforward way through this paradigm and exploiting reliable sensors for the measurement of the key PVs, together with algorithms for the detection of bad input data. 

An additional issue that may arise in FDI systems that would be considered as lasting applications is the performance degradation. This problem occurs for both model-based and model-free approaches.

As previously mentioned, the proposed FDI framework exploits PCA. As explained in Section 2.3, the application of the ANOVA test for the selection of the PCs provides a robust criterion which does not depend on subjective design choices. In addition, the ANOVA test requires an average computational effort (see Section 2.3.1) when compared to the other methods described in Section 2.3 and it is not influenced by the dimension of the dataset.

## 3. Results and Discussion

In the present section, some results on the proposed FFC for FDI are reported. The NMSC is considered as case study. All the reported results were obtained in the MATLAB environment (see Section 2.5 for details on the computational framework).

### 3.1. ANOVA Test PCs Selection Results

The PCs selection method based on the ANOVA statistical test was applied to historical data of the NMSC described in Section 2.1. The compressor variables that have been considered in the proposed results are a subset of the PVs reported in Table 1. The considered PVs are N_2_ flow (PV1), position of the IGV (PV2), position of the vent valve (PV4), compression ratio (PV9), polytrophic efficiency (PV10), power consumption (PV12), temperature (PV13), and vibrations (PV14 and PV15). The multishaft centrifugal compressor dataset considered is composed of 2500 samples for each of the nine variables; each sample refers to a time interval of five minutes. The process data are scaled to the zero mean and unit variance. In order to illustrate the ANOVA procedure, eigenvalues of matrix A (see Section 2.2) are summarized in Table 4.

**Figure 7:**
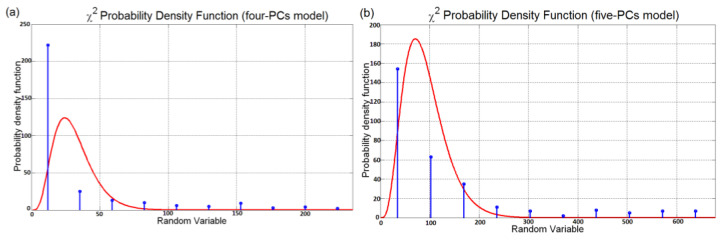
ANOVA Test PCs Selection Results: histogram of the reconstruction error relative to the four-PCs model (**a**) and five-PCs model (**b**).

The number l of the PCs is to be augmented up to four in order to obtain the three assumptions of the ANOVA test fulfilled for the first time. At this point, in order to test whether further increasing the subspace dimension could be beneficial, the ANOVA test is repeated considering five PCs. Figure 7 depicts the PDF computed on the reconstruction errors associated to the four-PCs model and to the five-PCs model, respectively. From their inspection, it can be stated that the first hypothesis of the sum of the square errors is fulfilled and, consequently, that, as discussed in the previous section, the reconstruction errors follow a normal distribution with zero mean and unit variance.

**Figure 8:**
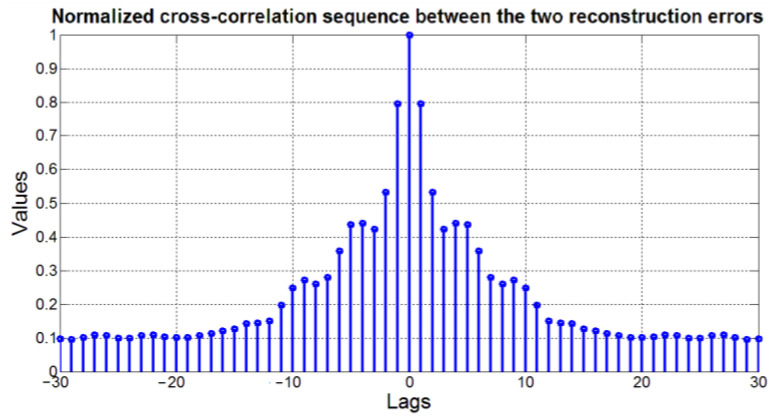
ANOVA Test PCs Selection Results: normalized cross-correlation sequence of the reconstruction errors.

**Figure 9:**
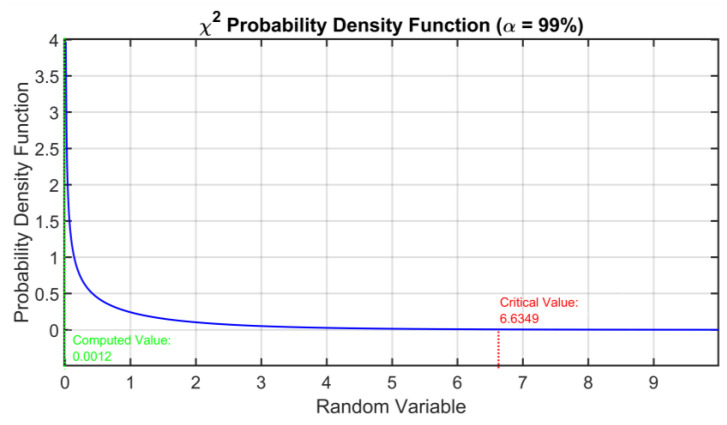
ANOVA Test PCs Selection Results: χ^2^ probability density function used in Bartlett’s test.

**Figure 10:**
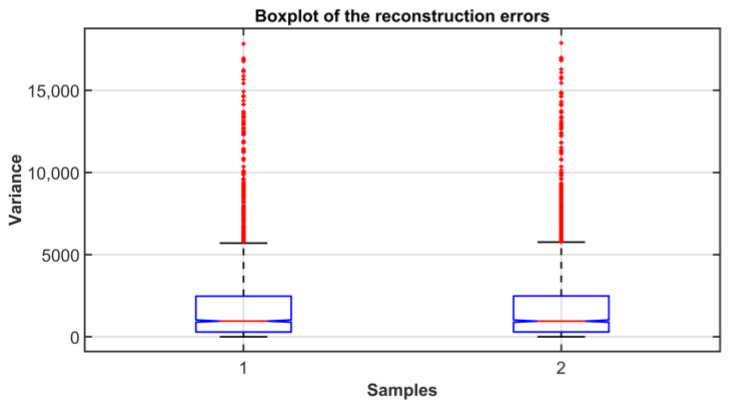
ANOVA Test PCs Selection Results: boxplot of the reconstruction error.

**Figure 11:**
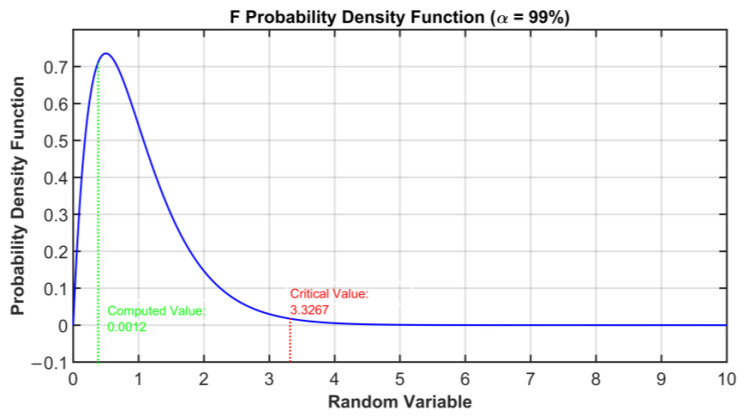
ANOVA Test PCs Selection Results: F probability density function used in the ANOVA test.

**Figure 12:**
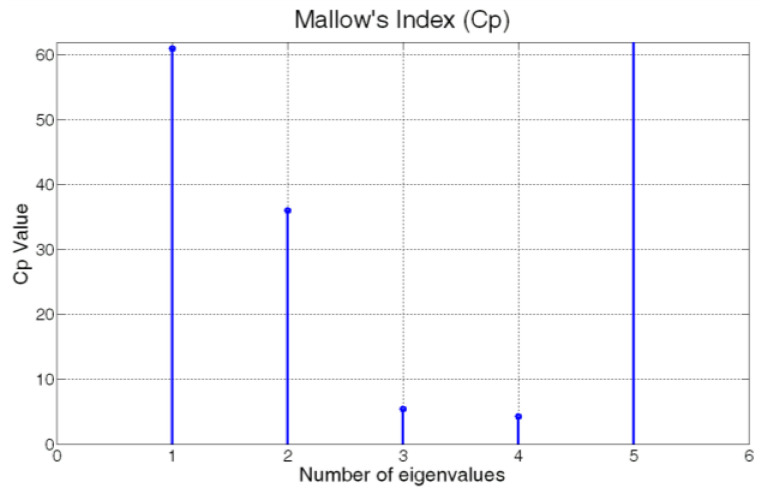
ANOVA Test PCs Selection Results: *C_p_* Mallows Index.

Figure 8 shows the cross-correlation sequence of the reconstruction errors of the two considered models. As previously explained, from the assumption of normal distribution, the independence condition can be thus verified since the two error signals proved to be uncorrelated. Finally, the homoscedasticity property is checked using Bartlett’s test (see Section 2.3). In Figure 9, the resulting distribution is shown. Table 5 summarizes the results of the test. A further validation of the equality of the variances of the reconstruction errors is obtained by inspecting the boxplot representation of Figure 10. From the ANOVA test, it can be concluded that the model with four eigenvalues is adequate for the reconstruction of the original variables (see Table 6 and Figure 11). In Table 7 and Figure 12, the *C_p_* Mallows index is reported as well which is in agreement with the result suggested by ANOVA. As can be noted, the *C_p_* Mallows index associated to the model with four eigenvalues is the lowest one (see bold value in Table 7) and consequently this model is recommended to be the most adequate model.

Eigenvalues in bold characters in the Table 4 are the ones chosen by the proposed method based on the analysis of variance test. If “manual” eigenvalue inspection would have been performed, different subjective selections could have been possible; for example, if a criterion based on the inspection of the gap between consecutive eigenvalues was adopted, then the resulting number of PCs could be five, but also just two (see Table 4). If two PCs are chosen, a not-satisfactory reconstruction of the variable is achieved; on the other hand, if five PCs are taken into account, overfitting negatively affects the problem solution. By choosing four PCs, as calculated by the procedure based on the ANOVA test, we instead obtain a good trade-off between accurate reconstruction of the input data and the problems caused by overfitting.

**Figure 13:**
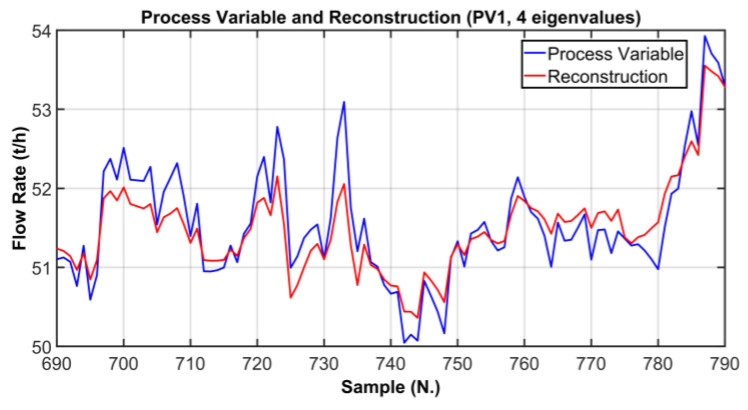
ANOVA Test PCs Selection Results: comparison between the original PV and the reconstructed PV (PV1, 4 eigenvalues).

**Figure 14:**
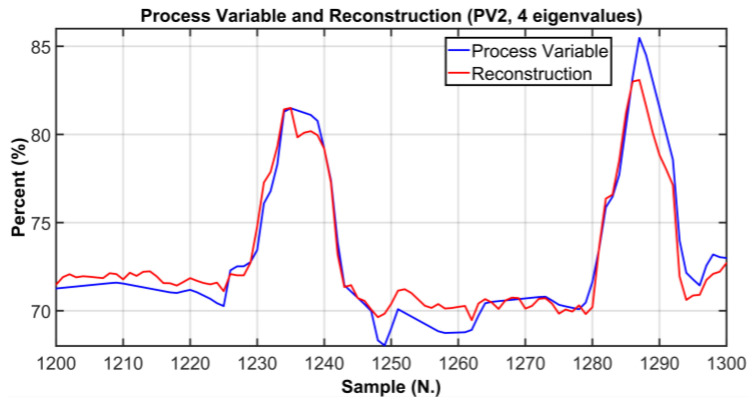
ANOVA Test PCs Selection Results: comparison between the original PV and the reconstructed PV (PV2, 4 eigenvalues).

**Figure 15:**
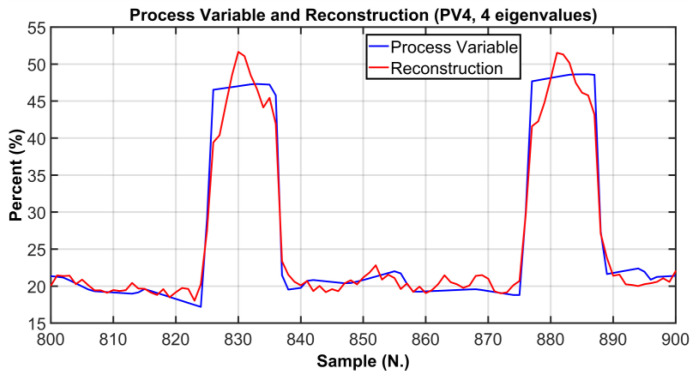
ANOVA Test PCs Selection Results: comparison between the original PV and the reconstructed PV (PV4, 4 eigenvalues).

In order to verify whether the selected number of PCs is adequate to correctly explain the original process variables, a comparison between original process variables and the reconstructed ones was performed. In Figure 13, Figure 14 and Figure 15, graphical comparisons of the most significative variables are reported. The original PVs are represented by blue line while the reconstructed ones are shown by a red line; in order to provide a significant graphical detail, only 100 samples are plotted. As it can observed, a satisfactory reconstruction is obtained. Table 8 reports the Root Mean Square Error (RMSE) between the original PVs and the reconstructed ones on the reported samples. In addition, in order to provide a more intuitive metric for reconstruction procedure evaluation, the RMSE is expressed also as a percent of the typical variation range of the considered variables. As can be noted in Table 8, the percent metric never exceeds the value of 11 percent of the range of variation considered.

### 3.2. Comparison between the ANOVA Test PCs Selection Method and Other Methods

**Figure 16:**
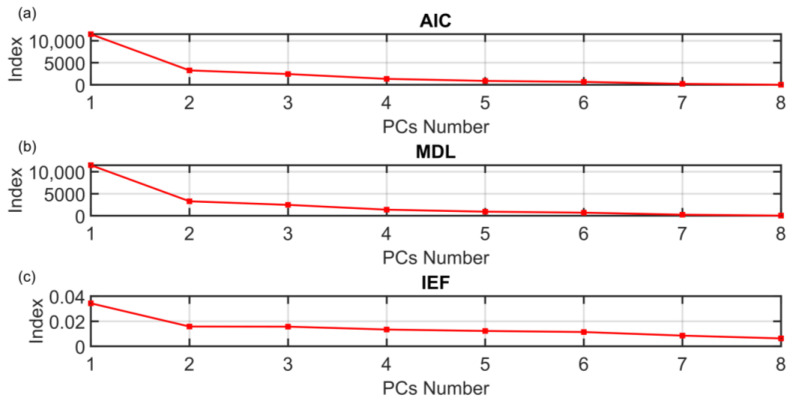
(**a**) AIC, (**b**) MDL and (**c**) IEF PCs Selection Results.

**Figure 17:**
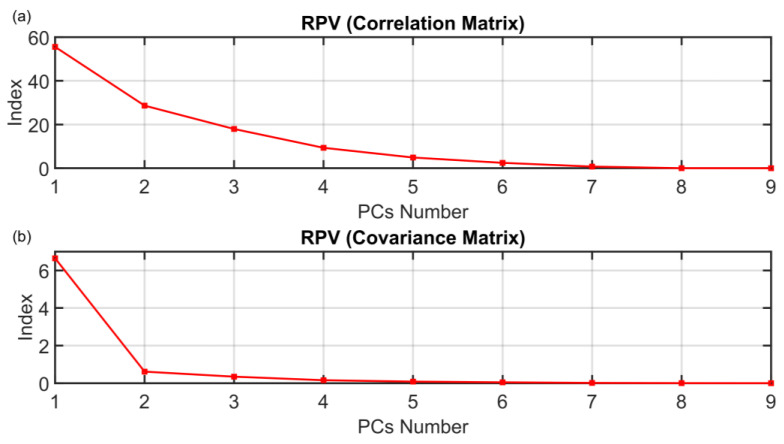
RPV (Correlation Matrix (**a**) and Covariance Matrix (**b**)) PCs Selection Results.

**Figure 18:**
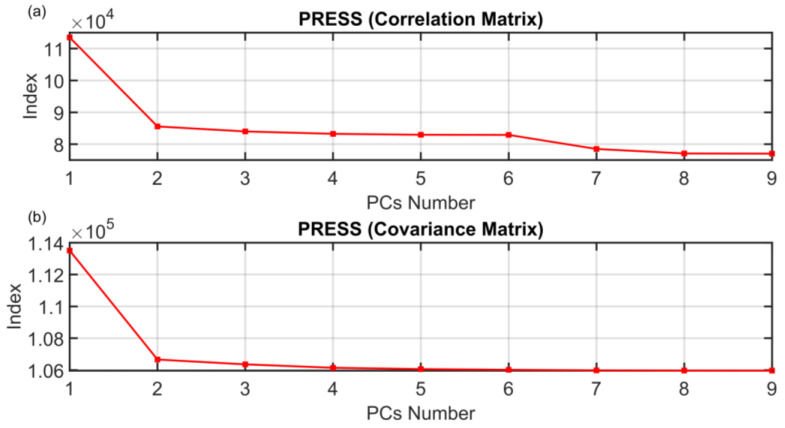
PRESS (Correlation Matrix (**a**) and Covariance Matrix (**b**)) PCs Selection Results.

**Figure 19:**
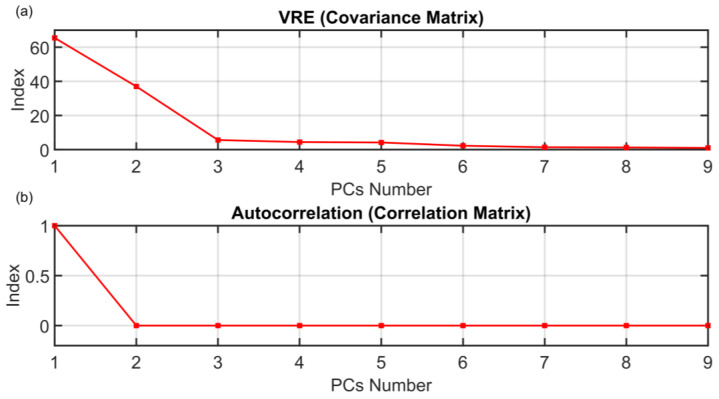
(**a**) VRE (Covariance Matrix) and (**b**) AC (Correlation Matrix) PCs Selection Results.

**Figure 20:**
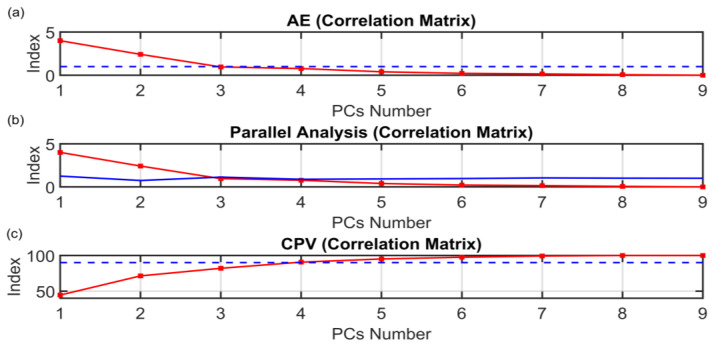
(**a**) AE, (**b**) Parallel Analysis and (**c**) CPV (Correlation Matrix) PCs Selection Results.

The motivation for the development of the ANOVA method presented in Section 3.1 is to find a rigorous way to determine the dimension of the PCs subspace when approaching FDI problems with PCA techniques. The results on the PCs selection of the ANOVA method presented in Section 3.1 are here compared with some of the most common techniques proposed in the literature. The following figures show the behaviors of the indices when varying the number of selected PCs (dots). The results of the other methods are not as encouraging as the results of the ANOVA test. As it can be seen from Figure 16, the monotonic behavior of the AIC, MDL and IEF methods prevents an acceptable solution from being obtained. Figure 17 and Figure 18 report the results obtained applying RPV and PRESS methods to both correlation and covariance matrices. With regard to the correlation matrix case (Figure 17a and Figure 18a), a few aspects can be pointed out. In Figure 17a, it can be observed that the RPV index applied to correlation matrix is monotonically decreasing and consequently it is impossible to find a “knee” in the curve. On the other hand, a first check of the PRESS indicator applied to correlation matrix (see Figure 18a) may infer the choice of four PCs in the model, but this solution appears to be ambiguous and subjective since the curve has a minimum point in correspondence of the ninth PC. With regard to the covariance matrix case (see Figure 17b and Figure 18b), these methods suggest three PCs. The same result is provided by the VRE method (see Figure 19a). However, it was verified that this dimension of the PCs subspace is not adequate to reconstruct the monitored variables: the coefficient of determination R^2^ is too low (not reported for brevity). Among other methods, the AC method (see Figure 19b) improperly suggests retaining only one eigenvalue in the model. Finally, the AE, PA and CPV methods suggest to use four eigenvalues as computed also through the ANOVA test (see Figure 20). Nevertheless, the AE and PA methods may depend on the number of the dataset samples, while in the CPV method, different choices of the arbitrary threshold may modify the solution. The comparison results are summarized in Table 9. On the other hand, the ANOVA test method is not influenced by the dimension of the dataset; it requires a medium computational effort and it can be applied to both the covariance and the correlation matrix. Finally, its major benefits can be stated to be its reliability, its objectiveness and the uniqueness of the proposed solution.

### 3.3. Results on Fuzzification and Cluster Analysis

In order to show how the designed fuzzification and Cluster Analysis procedures work (see Section 2.4.1 and Section 2.4.2), an example is proposed here. Consider the case of just two operative conditions: NOC, i.e., ID #1 in Table 3, and a fault on the N_2_ mass flow sensor of the NMSC first section (PV1), i.e., fault ID #2. In this case, implementing the previously described and tested PCA procedure, four PCs are selected and 255 SPEs combinations are considered. The most significant SPE components are reported in Table 10. The SPEs associated to the fault at issue show a degree of membership (of the Membership Function) close to 1 (see bold red values in Table 10), while the other components show a degree of membership of less than 0.5.

**Figure 21:**
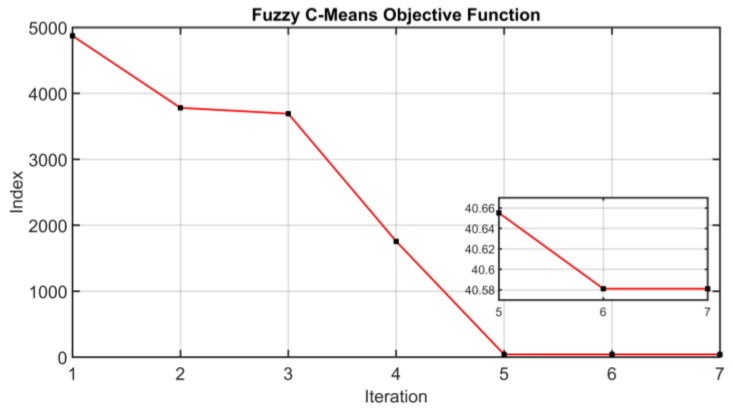
Results on Fuzzification and Cluster Analysis: objective function of the Fuzzy C-Means algorithm.

As explained in Section 2.4.2, the centroids are then obtained through the minimization of the objective function defined for the Fuzzy C-Means algorithm. The behavior of the considered objective function is reported in Figure 21. The convergence of the algorithm is obtained in seven iterations. 

Considering all the operative conditions reported in Table 3, the described procedure contributes to the achievement of all the fault prototypes mentioned in Section 2.4.2. 

### 3.4. Results on the Validation of the Clustering Consistency

From the application of the offline procedure, eighteen fault prototypes were classified (see Table 3) and their consistency was validated using the JM method. In this section, an example to show the validity of the exploited JM method for clustering detection and consistency validation (see Section 2.4.2) on the NMSC case study is provided. The PVs mentioned in Section 3.1 and Section 3.2 were selected, thus considering nine variables. After interviewing the operators, it was possible to select two datasets of the process data in correspondence of two process faults of the machine. In general, this information is not available, and if no procedure concerning the validation of the cluster generation approach is employed, there is no certainty for each selected dataset of the number of different process conditions. Given a set of historical data, the number of different conditions included in the data is not known with certainty. To overcome this problem, the JM procedure is exploited. When using JM in cluster consistency validation, in order to accept the outcome of the cluster generation method, it is necessary that the JM distances between the computed clusters are such that the separation between them is well defined. In the example considered here, the whole dataset refers to three different operative conditions of the compressor (no faults, process fault #1, and process fault #2, see Table 3): three different clusters are expected as the result of the procedure. Thus, the final expected result from the application of the JM procedure is that the dataset can be clustered into three different clusters. 

**Figure 22:**
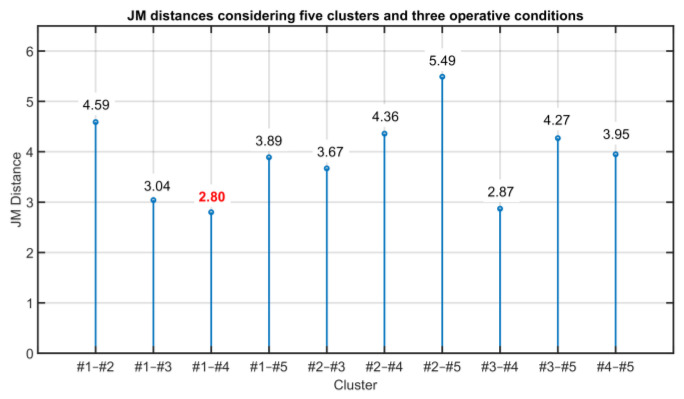
Results on the Validation of the Clustering Consistency: JM distances considering five clusters and three operative conditions. Shown in red is the minimum JM distance.

The initial number of clusters can be derived from the information on the process but it can be also assumed empirically. In case the initial guess is too high, the JM procedure needs many iterations to converge to the final number of clusters; on the other hand, if the initial guess is too low, at the first iteration, no reduction in the number of clusters is suggested. In this case, a greater number of clusters should be used as the initial guess. To test the proposed procedure, it was chosen to initially assume five different clusters. Since the data matrix under study is composed of *n* = 9 variables, to speed up the procedure, instead of considering the JM distance between the single variables for each pair of clusters, the JM distance between pairs of clusters is computed as the sum of the JM distances between all variables. In Figure 22, the JM distances between cluster pairs are summarized.

The next step of the procedure aims to check for the possible merging of the clusters. The two clusters in correspondence of the minimum JM distance (see the bold red value in Figure 22) could be merged in a single cluster if the following requirement is satisfied. Considering the maximum value reachable by JM distance ($JM_{ij}$) equal to $\sqrt{2}$ (see [89] for further details), and considering the number of the variables (nine), the clusters in correspondence of the minimum value of the JM metric can be merged if the distance $JM_{ij}$ between each of these two clusters and the other clusters satisfies

(14) $$JM_{ij}\leq \sqrt{2}\cdot n\cdot k,$$

where *k* is a tolerance threshold usually in the range 0.03–0.06 (see [89] for further details). Applying Equation (14) to the considered case study, the following equation holds:

(15) $$JM_{ij}\leq 0.5.$$

Thus, considering clusters #1 and #4 which are characterized by the minimum JM distance, the distances of cluster #1 and #4 to the other clusters are compared and their difference is computed (see Table 11).

**Figure 23:**
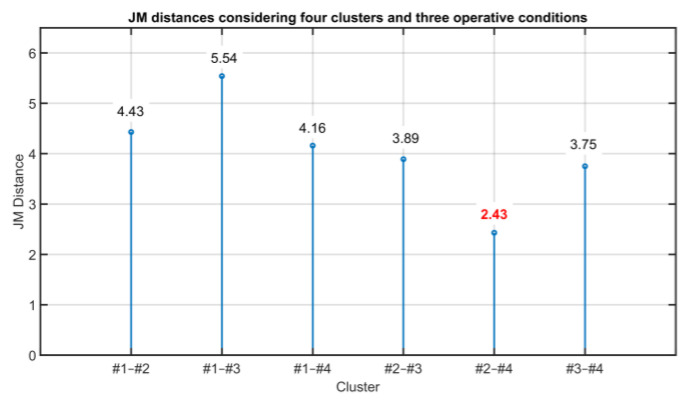
Results on the Validation of the Clustering Consistency: JM distances considering four clusters and three operative conditions. Shown in red is the minimum JM distance.

**Figure 24:**
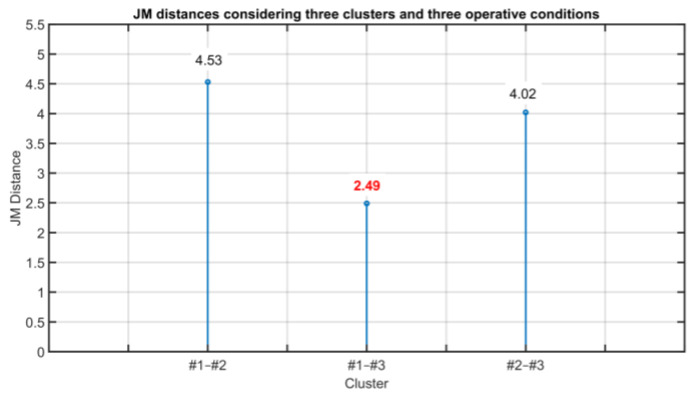
Results on the Validation of the Clustering Consistency: JM distances considering three clusters and three operative conditions. Shown in red is the minimum JM distance.

As it can be noted in Table 11, clusters #1 and #4 satisfy the requirement and can be merged together. The procedure continues by computing the JM distances considering the four clusters, obtaining Figure 23. In Figure 23, the pair of clusters with the minimum value of the JM distance is (#2, #4) (see the bold red value in Figure 23). The JM distances between them and the other clusters are reported in Table 12. As it can be noted from Table 12, clusters #2 and #4 satisfy the requirement and can be merged together. The procedure continues by computing the JM distances considering three clusters, obtaining Figure 24. In Figure 24, the pair of clusters with the minimum value of the JM distance is (#1, #3) (see the bold red value in Figure 24). The JM distances between them and the other cluster are reported in Table 13.

As can be noted in Table 13, the difference between the two distances is greater than 0.5 and the condition for cluster aggregation does not hold. As further validation of this statement, the two clusters are considered and the JM distance is computed; the resulting distance is equal to 3.20. Since the minimum value of the JM distances is lower than the minimum value of the JM distances (equal to 2.80), the last reduction of the number of clusters cannot be accepted. Thus, at the end of the procedure, three clusters are suggested.

The reported results on the validation method of the clustering consistency show the validity of the proposed methodological approach.

### 3.5. Results on NMSC FDI

After showing the results regarding the correct sizing of the PCs subspace and the fault prototypes, the results on the performance of the developed FDI module and the effectiveness of the classification algorithm in diagnosing the correct fault are illustrated here. In particular, the fouling of the compressor stage is first proposed. This fault is of particular interest because it is generally not easily diagnosed. In fact, in this type of fault, which we have termed process fault and which affects many variables, often, the individual variations are not very evident. As additional results, the system performance in case of both instrument single and multiple faults are reported.

#### 3.5.1. Process Fault: Fouling of the Compressor Stage

**Figure 25:**
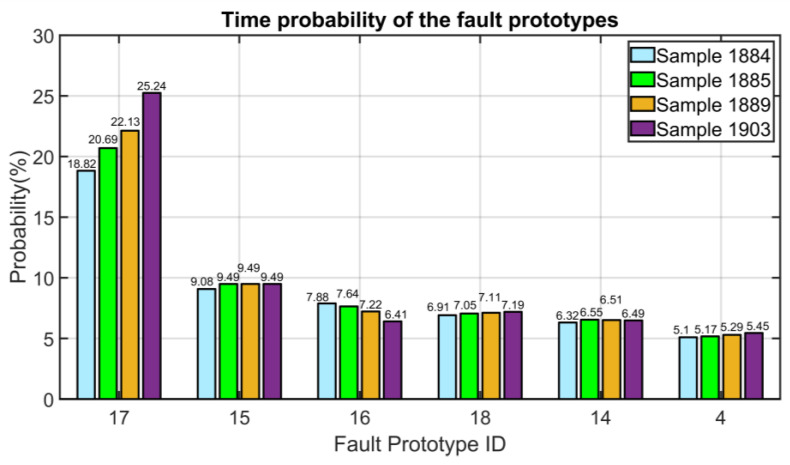
Results on NMSC FDI: fouling of the compressor stage (time probability of the most significant fault prototypes).

A dataset of 2500 samples with sampling time equal to 5 min is exploited for the validation of the designed FDI system. The FDI system exploits the results described in Section 3.1, Section 3.2 and Section 3.4; nine PVs are chosen and the ANOVA test provides four PCs. In the first instants of the exploited dataset, no faults are detected by the proposed algorithm and the “no fault” (ID #0, see Table 3) probability is 100%. The results at each instant are shown by a histogram. At Sample Instant 1884, when the effects of the fouling begin to be relevant, the classification algorithm correctly identifies the fouling. In Figure 25, the faults that have the highest probability are shown while the faults with a probability lower than 5% are neglected. Here, the light blue bars refer to Sample Instant 1884: the faults with ID #17, #15, #16, #18, #14, #4 (see Table 3) are characterized by a probability higher than 5%. The probability is computed within the proposed FFC module (see Section 2.4). At the considered sample instant (1884), the fault with ID #17 (fouling, see Table 3) is characterized by the highest probability. In the subsequent instants, the probability associated to the fault with ID #17 increases (see green, yellow and purple bars in Figure 25) while the probability associated to the other most probable faults remains lower than 10%. The proposed example shows how the proposed FDI system is capable to detect and isolate the fault; in the real plant, more than one month was needed to the operators and engineers to isolate the fault, so the plant operated in inefficient conditions for a significant time period.

#### 3.5.2. Instrument Single Fault: Error on Thermocouple Relative to the First Stage Bearing (PV13)

**Figure 26:**
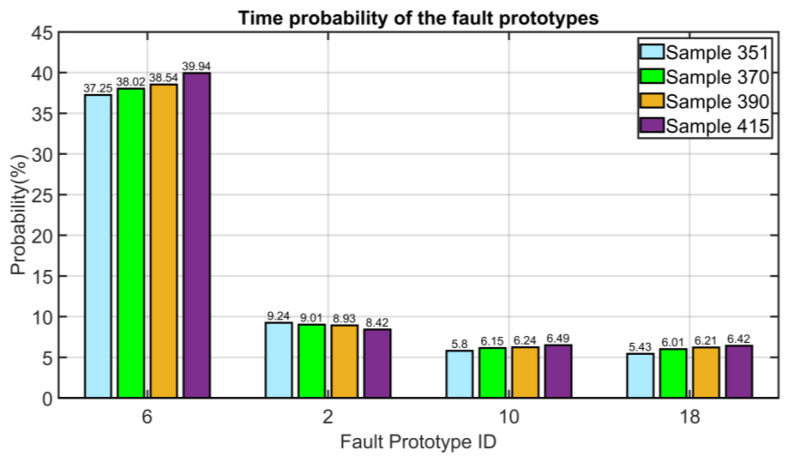
Results on NMSC FDI: error on thermocouple relative to the first stage bearing (time probability of the most significant fault prototypes).

An instrument single fault is considered in this section. An error on thermocouple relative to the first stage bearing (PV13) is simulated at Sample 351. A step variation with a magnitude of 20% of the NOC value of the PV is simulated. As can be noted in the histogram plotted in Figure 26, the FFC module assigns a high probability to the fault with ID #6 which corresponds to the simulated fault (see Table 3). In the depicted subsequent sampling instants (370, 390, and 415), the probability computed by the FFC module associated to the fault with ID #6 increases, while the probability associated to the other most probable faults remains lower than 10% (the faults with a probability lower than 5% are neglected).

#### 3.5.3. Instrument Multiple Faults: Simultaneous Error on the Vibration’s Measurements (PV14 and PV15)

**Figure 27:**
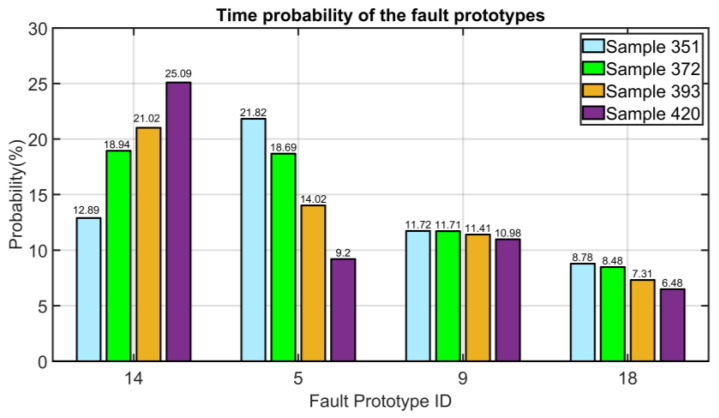
Results on NMSC FDI: simultaneous error on the vibration measurements (time probability of the most significant fault prototypes).

Instrument multiple faults are proposed in this section. In particular, to identify the simultaneous malfunction in the horizontal and in the vertical sensors (vibration measurements), two additive steps are simulated at Sample 351. At this sample instant, as can be observed in Figure 27 (see light blue bars), the FFC module erroneously assigns the higher probability to the fault with ID #5 which corresponds to the single fault on the vertical vibration measurement (see Table 3). However, in the next steps, the configurations of the SPEs approach the configuration relative to the correct centroid (ID #14); in fact, after 21 steps, we obtain the probability computation depicted through green bars in Figure 27, which reports that the fault with ID #14 is now the fault with the highest probability (18.94%). The fault with ID #14 is detected and isolated by the proposed algorithm and its probability computed by the FFC module increases up to 25.09% at Sampling Instant 420. Despite the fact that, in the first samples, the FDI system had difficulties identifying the true fault, a few samples after, it sent a correct message. The delay exhibited in correctly identifying the fault is limited and, considering the behavior of the process, can be accepted. In addition, in this case, the initial misidentification of the fault problem was not completely erroneous since the measurement of the vertical vibration was effectively present.

## 4. Conclusions

The present paper proposes the application of an FDI system on a Nitrogen MultiShaft Compressor. The FDI method exploits Principle Components Analysis, Cluster Analysis and Pattern Recognition. 

The exploited method focuses on the determination of the dimension of the PCs subspace through the ANOVA (ANalysis Of VAriance) test. Cluster Analysis and Pattern Recognition techniques were integrated in a Fuzzy Faults Classifier (FFC) module and applied in order to tackle complexity issues caused by the high number of PVs to be taken into account in the considered case study. In addition, a probability computation of each different fault at each sample instant is provided by the designed FFC module. The output of the FDI system is given in terms of fault probability and the faults characterized by a higher probability are notified. These features provide the user with more comprehensive information.

Instrument single and multiple faults are associated to the compressor sensors and actuators; in addition, the FDI system is able to diagnose process faults. Examples of process faults are the fouling of the compression stages and the breaking of the thrust bearing. The obtained results, associated to each single phase of the FDI method, show the reliability and the effectiveness of the method. From a practical point of view, it is proven that the FDI system based on FFC module can infer the increase in plant safety while reducing costs.

Future work will be focused on the application of the method to other fields and to the application of other FDI methods to the proposed case study.

## Figures and Tables

**Table 1:** Most common NMSC faults.

Fault Description	Time Dependency
Second section N_2_ mass flow (sensor)	incipient fault abrupt fault
High pressure N_2_ mass flow (sensor)	incipient fault abrupt fault
First section N_2_ mass flow (sensor)	incipient fault abrupt fault
Third stage IGV (positioner)	incipient fault intermittent fault abrupt fault
First stage IGV (positioner)	incipient fault intermittent fault abrupt fault
Fouling of the first stage of the NMSC	incipient fault
Breaking of the thrust bearing relative to the first stage	incipient fault

**Table 2:** NMSC PVs.

PV#	PV Description	Measurement Unit
PV1	N_2_ mass flow through the first section of the NMSC	[t/h]
PV2	N_2_ Positioner of the IGV relative to the first and second stage of NMSC	[%]
PV3	Positioner Feedback of IGV position relative to the NMSC first and second stage	[%]
PV4	Vent position at the entrance of first section of NMSC	[%]
PV5	N_2_ mass flow through the second section of NMSC	[t/h]
PV6	Positioner of the IGV relative to the third stage of NMSC	[%]
PV7	Feedback of IGV position relative to the third stage of NMSC	[%]
PV8	Throttle valve position relative to inlet high pressure nitrogen gas	[%]
PV9	Compression ratio of the first stage of NMSC	[-]
PV10	Polytrophic efficiency of the first stage of NMSC	[-]
PV11	N_2_ mass flow from the head of the high-pressure column	[t/h]
PV12	Power consumption by NMSC	[kW]
PV13	Thrust bearing temperature of the first shaft	[°C]
PV14	Horizontal vibrations of the first shaft of NMSC	[μm]
PV15	Vertical vibrations of the first shaft of NMSC	[μm]
PV16	Throttle valve position relative to inlet high pressure N_2_ gas	[%]
PV17	N_2_ temperature at the inlet of the 5th stage of the NMSC	[°C]
PV18	N_2_ pressure at the inlet of the 5th stage of the NMSC	[bar]
PV19	N_2_ pressure at the exit of the heat exchanger used in the 5th stage of the NMSC	[bar]
PV20	N_2_ temperature at the exit of the heat exchanger used in the 5th stage of the NMSC	[°C]
PV21	Thrust bearing temperature of the shaft	[°C]
PV22	Horizontal vibrations of the 5th shaft of NMSC	[μm]
PV23	Vertical vibrations of the 5th shaft of NMSC	[μm]
PV24	Thrust bearing temperature of the shaft	[°C]
PV25	Thrust bearing temperature of the shaft	[°C]
PV26	N_2_ temperature at the exit of the 5th stage of the NMSC	[°C]
PV27	H_2_O temperature at the exit of the heat exchanger used in the 5th stage of the NMSC	[°C]

**Table 3:** NMSC fault prototypes.

Fault Prototype ID	Fault Prototype Description
(1)	Absence of faults
(2)	Failure in the N_2_ mass flow sensor in the first section (PV1)
(3)	Error in the control of first stage IGV position (PV2)
(4)	Error in the horizontal vibration’s measurement (PV14)
(5)	Error in the vertical vibration’s measurement (PV15)
(6)	Fault in the thermocouple relative to the first stage bearing (PV13)
(7)	Simultaneous fault in the first section N_2_ mass flow sensor (PV1) and in the control of first stage IGV position (PV2)
(8)	Simultaneous fault in the first section N_2_ mass flow sensor (PV1) and in the horizontal vibration’s measurement (PV14)
(9)	Simultaneous faults in the first section N_2_ mass flow sensor (PV1) and in the vertical vibration’s measurement (PV15)
(10)	Simultaneous faults in the first section N_2_ mass flow sensor (PV1) and in the thermocouple relative to the first stage bearing (PV13)
(11)	Simultaneous faults in the control of first stage IGV position (PV2) and in the horizontal vibration’s measurement (PV14)
(12)	Simultaneous faults in the control of first stage IGV position (PV2) and in the vertical vibration’s measurement (PV15)
(13)	Simultaneous faults in the control of first stage IGV position (PV2) and in the thermocouple relative to the first stage bearing (PV13)
(14)	Simultaneous error in the vibration’s measurements (PV14 and PV15)
(15)	Simultaneous fault in the horizontal vibration’s measurement (PV14) and in the thermocouple relative to the first stage bearing (PV13)
(16)	Simultaneous fault in the vertical vibration’s measurement (PV15) and in the thermocouple relative to the first stage bearing (PV13)
(17)	Fouling of the first stage of the NMSC
(18)	Breaking of the thrust bearing relative to the first stage

**Table 4:** ANOVA Test PCs Selection Results: eigenvalues of matrix A.

Eigenvalue
**5687.9**
**4767.2**
**3767.0**
**2773.9**
2036.3
1416.8
996.1
800.9
245

**Table 5:** ANOVA Test PCs Selection Results: Bartlett’s Test.

Crucial Value	Calculated Value	Result
6.6349	0.0012	The assumption of equality of the variances of the reconstruction errors is fulfilled

**Table 6:** ANOVA Test PCs Selection Results: ANOVA test detail.

Degree of Freedom	Sum of Squares	Mean Squares	Computed F Value	Critical F Value	*C_p_* Mallows Index
p − 1	0.15 × 10^5^	3.67 × 10^3^	3.3267	0.3754	4.26
N − p	4.55 × 10^5^	1.55 × 10^3^			
N − 1	4.69 × 10^5^	1.57 × 10^3^			

**Table 7:** ANOVA Test PCs Selection Results: *C_p_* Mallows Index.

Number of Eigenvalues	*C_p_* Value
1	61.0314
2	35.9931
3	5.3742
**4**	**4.2606**
5	1128.8

**Table 8:** ANOVA Test PCs Selection Results: RMSE computation for the comparison between the original PVs and the reconstructed PVs (4 eigenvalues).

PV#	RMSE	% RMSE/Range
PV1	0.5148 t/h	6.8159%
PV2	4.2280%	4.7%
PV4	9.4586%	10.51%

**Table 9:** PCs Selection Results: comparison between different methods.

Method	PCs Number
AIC	No solution
MDL	No solution
IEF	No solution
PRESS_cov_	No solution
RPV_corr_	No solution
PRESS_corr_	Ambiguous
AC	1
RPV_cov_	3
VRE	3
ANOVA	4
AE_corr_	4
PA_corr_	4
CPV_corr_	4

**Table 10:** Results on Fuzzification: some of the 255 components of the SPEs associated to NOC and to the fault with ID #2.

Fault Prototype ID/SPE Component	1	2	3	4	5	6	7	8	9
NOC—ID #1	**2.49 × 10^−5^**	**9.96 × 10^−5^**	**1.32 × 10^−5^**	**4.87 × 10^−5^**	**2.02 × 10^−5^**	**1.28 × 10^−5^**	**1.42 × 10^−5^**	**4.80 × 10^−5^**	**9.04 × 10^−6^**
Fault—ID #2	**0.49**	**0.99**	**0.29**	**0.36**	**0.42**	**0.27**	**0.33**	**0.45**	**0.18**
**Fault Prototype ID/SPE Component**	**10**	**…**	**18**	**19**	**20**	**21**	**22**	**23**	**…**
NOC—ID #1	**0**	**…**	**9.52 × 10^−5^**	**0**	**9.14 × 10^−5^**	**9.94 × 10^−5^**	**8.51 × 10^−5^**	**0**	**…**
Fault—ID #2	**0.99**	**…**	**0.98**	**0.99**	**0.98**	**0.99**	**0.97**	**0.99**	**…**

**Table 11:** Results on the Validation of the Clustering Consistency: JM distances considering five clusters and three operative conditions (focusing on clusters #1 and #4).

Clusters	JM Distance		Difference
#1–#2	4.59	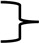	0.23 < 0.50
#4–#2	4.36		
#1–#3	3.04	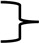	0.17 < 0.50
#4–#3	2.87		
#1–#5	3.89	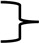	0.06 < 0.50
#4–#5	3.95		

**Table 12:** Results on the Validation of the Clustering Consistency: JM distances considering four clusters and two operative conditions (focusing on clusters #2 and #4).

Clusters	JM Distance		Difference
#2–#1	4.33	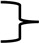	0.17 < 0.50
#4–#1	4.16		
#2–#3	3.89	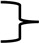	0.14 < 0.50
#4–#3	3.75		

**Table 13:** Results on the Validation of the Clustering Consistency: JM distances considering three clusters and three operative conditions (focusing on clusters #1 and #3).

Clusters	JM Distance		Difference
#1–#2	4.53	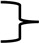	0.51 > 0.50
#3–#2	4.02		

## Data Availability

Not applicable.
